# Adhesins of Yeasts: Protein Structure and Interactions

**DOI:** 10.3390/jof4040119

**Published:** 2018-10-27

**Authors:** Ronnie G. Willaert

**Affiliations:** 1 Alliance Research Group VUB-UGent NanoMicrobiology (NAMI), IJRG VUB-EPFL NanoBiotechnology & NanoMedicine (NANO), Research Group Structural Biology Brussels, Vrije Universiteit Brussel, 1050 Brussels, Belgium; Ronnie.Willaert@vub.be or Ronnie.Willaert@uantwerpen.be; Tel.: +32-26291846; 2 Department Bioscience Engineering, University Antwerp, 2020 Antwerp, Belgium

**Keywords:** yeast adhesions, *Saccharomyces cerevisiae*, *Candida albicans*, *Candida glabrata*, the flocculation protein family, the epithelial adhesion family, the agglutinin-like sequence protein family, Flo proteins, Als proteins, Epa proteins

## Abstract

The ability of yeast cells to adhere to other cells or substrates is crucial for many yeasts. The budding yeast *Saccharomyces cerevisiae* can switch from a unicellular lifestyle to a multicellular one. A crucial step in multicellular lifestyle adaptation is self-recognition, self-interaction, and adhesion to abiotic surfaces. Infectious yeast diseases such as candidiasis are initiated by the adhesion of the yeast cells to host cells. Adhesion is accomplished by adhesin proteins that are attached to the cell wall and stick out to interact with other cells or substrates. Protein structures give detailed insights into the molecular mechanism of adhesin-ligand interaction. Currently, only the structures of a very limited number of N-terminal adhesion domains of adhesins have been solved. Therefore, this review focuses on these adhesin protein families. The protein architectures, protein structures, and ligand interactions of the flocculation protein family of *S. cerevisiae*; the epithelial adhesion family of *C. glabrata*; and the agglutinin-like sequence protein family of *C. albicans* are reviewed and discussed.

## 1. Introduction

Cellular adhesion is fundamental in many biological processes such as the development of multicellular organisms, as well as in a variety of contexts that are important to the life cycles of unicellular organisms [1,2]. Many fungi contain a family of cell wall glycoproteins, called “adhesins”, that confer unique adhesion properties [1]. These proteins are required for the interactions of fungal cells with each other (flocculation and filamentation) [1] and other cells such as in host–pathogen interactions [2]. The adherence of pathogenic yeasts to host tissues can occur at different sites in the human body. One of the potential adhesion targets is the glycocalyx, which represents the extracellular mesh of carbohydrate-rich biomolecules that are bound to cell membranes or secreted by cells into the external medium [3]. Adhesins with a lectin activity of pathogenic yeasts can target the glycoproteins of the glycocalyx or glycosylated host receptors [4,5].

The budding yeast *Saccharomyces cerevisiae* usually grows as a unicellular microorganism, but it can switch to a wide range of multicellular phenotypes, such as flocs, flors, filaments, and biofilms in response to changes in the environment and its genetic background [6]. These phenotypes enable *S. cerevisiae* to colonize various habitats, forage for nutrients, and escape unfavorable conditions [6,7,8,9]. Cell adhesion via cell-cell and/or cell-substrate interactions are necessary to form these multicellular structures.

Fungal infections are an extremely important health problem. About 1.2 billion of people are infected every year by fungi. Nevertheless, their contribution to the global burden of disease is largely unrecognized [10,11]. Some commensal *Candida* species belong to the human microbiome of healthy humans [12]. Impairment of the host immunity or the normal host microbiota can lead to *Candida* infection (candidiasis). Immunocompromised persons are very susceptible to fungal infections. Malnutrition, which encompasses both undernutrition and overnutrition, is responsible for an enormous health burden globally [13,14]. Results from disordered nutrient assimilation are also characterized by recurrent microbial infections and chronic inflammation, implying an underlying immune defect [15]. Defects in both the innate and adaptive arms of the immune system have been consistently demonstrated in undernourished children [16,17]. Undernourished children principally die of common infections [18,19]. Infections are also more common and severe in people with obesity [20]. The increased susceptibility of the malnourished host to bacterial, mycobacterial, and fungal infections is supported by data from clinical and animal studies [19,21]. Well-documented microbial infections include respiratory, gastrointestinal, and systemic candidiasis cases.

Five species of *Candida* (*C. albicans*, *C. glabrata*, *C. parapsilosis*, *C. tropicalis*, and *C. krusei*) account for more than 90% of all of the diagnosed cases of candidemia and invasive candidiasis, but their relative frequency varies depending on the population involved, geographical region, previous anti-fungal exposure, and patient age [22]. *C. albicans* is the most common etiological agent of candidiasis [23,24]. *C. glabrata* and other non-*albicans Candida* species are nowadays considered as emerging opportunistic organisms, since they represent an increasing number of fungal infections, which depends on the resistance of these yeasts to several antimicrobial agents [25,26]. *C. glabrata* is ranked second in isolation frequency among the *Candida* infections, and together with *C. albicans*, they are responsible for approximately 65–75% of all systemic candidiasis [27].

*Candida* adhesion to host cells (especially epithelial cells) represents the onset of infection, which is next followed by invasion. The strategies of *C. albicans* and *C. glabrata* to attach and invade into the host cells, obtain nutrients, and evade the host immune response show significant differences [28]. *C. albicans* follows an aggressive strategy to subvert the host response and obtain nutrients for its survival; *C. glabrata* evolved a strategy that is based on stealth, evasion, and persistence, without causing severe damage.

Despite the intensive research on yeast adhesion and unraveling the molecular mechanisms of adhesion over the last 30 years, only a few adhesin structures have been solved. Therefore, this review focuses on these adhesin protein families: the flocculation protein family of *S. cerevisiae*, the epithelial adhesin protein family of *C. glabrata*, and the agglutinin-like sequence protein family of *C. albicans*. The protein architectures of these yeast adhesins are compared based on the described architectures in the Pfam database. Next, the ligand–adhesin interaction mechanism is explained based on the protein structures of the N-terminal domains, which account for the adhesion domains of the adhesin. Finally, cellular adhesion mechanisms are discussed, i.e., cell-cell binding based on *S. cerevisiae*–lectin–flocculation interaction, cell-cell binding based on *S. cerevisiae*–Flo11p interaction, *C. albicans*–Als protein interactions, and *C. glabrata*–Epa protein interactions.

## 2. The Structure of Yeast Adhesins

### 2.1. Architectures of Yeast Adhesins

#### 2.1.1. The *S. cerevisiae* Flocculation Protein Family

The Flo adhesin protein family of *S. cerevisiae* can be subdivided into two groups [4]. The first group of proteins is encoded by genes, including *FLO1*, *FLO5*, *FLO9,* and *FLO10*. The gene products of *FLO1, FLO5, FLO9,* and—to a lesser extent—*FLO10* [9], promote cell-cell adhesion and form multicellular clumps (flocs), which sediment out of solution and therefore are called flocculins [10]. Their genes share considerable sequence homology. *FLO10* and *FLO11*, but not *FLO1*, also promote both adhesion to agar and plastic, and filamentation. The second group of the Flo family, which includes Flo11p, Fig2p, and Aga1p, has a domain structure that is similar to that of the first group, but with quite unrelated amino acid sequences [29]. Flo11p also promotes cell–cell adhesion, but does this only weakly [9]. Flo11p is mainly required for diploid pseudohyphal formation, haploid invasive growth [6,11], and biofilm formation [30]. N-Flo11p does not bind to mannose, which is in contrast to the other Flo proteins. However, N-Flo11p can interact with N-Flo11p (homotypic adhesion ability), explaining the weak flocculation characteristic [14,15]. Fig2p and Aga1p both contain repeated, conserved WCPL and CX4C domains [31]. These proteins are involved in mating and are induced by the pheromones from cells of the opposite mating type [32,33,34,35,36]. Fig2p normally functions in the adhesion of mating cells. When overexpressed, Fig2p and Flo11p can function in mating, invasion, filamentation, and flocculation [29]. The ability of Flo11p to supply Fig2p function in mating depends on its intracellular localization to the mating projection. During mating, heterotypic interactions between Aga1p and Fig2p and a homotypic interaction between Fig2p and Fig2p can occur [31].

**Figure 1:**
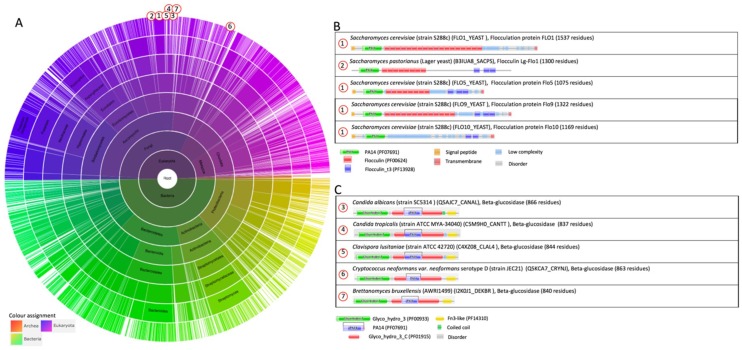
The family PA14 (PF07691). (**A**) Sunburst phylogenetic representation of the PA14 domain family. (**B**) Currently (July 2018), the protective antigen (PA) domain is present in 499 architectures distributed over the superkingdom *Bacteria* (1573 sequences, 701 species), *Eukaryota* (1565 sequences, 379 species), and *Archaea* (18 sequences, 16 species). (**B**) Indicated domains are the PA14 domain (PA14, PF07691) [24]; the “Flocculin type 3 repeat” (Flocculin_t3, PF13928) that is found in Flo9 close to its C-terminus, and in a number of other *Saccharomyces* proteins [1]; and the “Flocculin repeat” (Flocculin, PF00624) that is rich in serine and threonine residues [2]. (**C**) Architectures for β-glucosidase that contain a PA14 domain illustrated for the pathogenic yeast *C. albicans*, *C. tropicalis*, *Clavispora lustinae*, and *Cryptococcus neoformans*; and *Brettanomyces bruxellensis*. Indicated domains are the “Glycosyl hydrolase family 3 N terminal” domain (Glyco_hydro_3 (PF00933) [30], the PA14 domain (PA14, PF07691) insert in “Glyco_hydro_3_C” (Glycoside hydrolase family 3, PF01915), and the “Fibronectin type III-like” domain (Fn3-like, PF14310) that is often found in association with “Glycoside hydrolase family 3” (PF00933, PF01915) [38]. Its function is unknown. The graphics were generated with Pfam version 31.0 [37].

**The PA14-Flo proteins architecture.** These adhesins have a modular configuration that is composed of three domains: the N-terminal, central, and C-terminal domain [37] (Figure 1 B). The amino-terminal secretory sequence (signal peptide) is removed when the protein moves to the plasma membrane through the secretory pathway [17]. The C-terminal domain contains a glycosylphosphatidylinositol (GPI) attachment signal sequence. The adhesins are covalently linked to the non-reducing end of β-1,6-glucans of the cell wall via the GPI remnant [18,19,20,21,22,23].

The N-terminal domain of the Flo proteins (including Lg-Flo1p) in this group contains the PA14 domain (Pfam entry PF07691; https://pfam.xfam.org/) [24] (Figure 1). This PA14 domain family was discovered based on the sequence analysis of an insert in a bacterial enzyme domain that showed homology with several protein sequences from other bacteria as well from eukaryotic organisms [24]. The insert is a 14-kDa region of PA_20_, which is a fragment of the protective antigen (PA) from anthrax toxin [25]. The presence of a calcium-dependent carbohydrate-binding pocket is a common element in the PA14 domain family [26,27]. This PA14 lectin domain in Flo1p, Flo5p, Flo9p, and Flo10p is responsible for cell–cell interaction and the formation of yeast flocs. The presence of this functional domain in the N-terminal domain was originally discovered by performing the following two experiments: the expression of a truncated Flo1p with a deleted PA domain (deletion of amino acids 50–278) resulted in non-flocculating cells [23]; and the replacement of the N-terminus of Flo1p by the corresponding region of Lg-Flo1p resulted in the conversion of the Flo1 flocculation phenotype to the NewFlo flocculation phenotype [28]. The PA14 domain can be involved in carbohydrate recognition or carbohydrate metabolism as part of a large number of enzymes, adhesins, and toxins [24,25]. The PA domain is present in 499 architectures distributed over the superkingdom *Bacteria* (1573 sequences, 701 species), *Eukaryota* (1565 sequences, 379 species), and *Archaea* (18 sequences, 16 species) (Figure 1A) (Pfam version 31, [38]). Outside the family *Saccharomycetaceae*, PA14 is mostly included in the architectures for β-glucosidase of yeasts, i.e., PA14 is included in the “Glycosyl hydrolase family 3 N terminal” domain (Glyco_hydro_3, Pfam entry PF00933) [30]. Examples are shown in Figure 1C: β-glucosidase architecture in the pathogenic yeasts *C. albicans*, *C. tropicalis*, *Clavispora lustinae*, and *Cryptococcus neoformans*, and the yeast *Brettanomyces bruxellensis*, which is involved in the spontaneous fermentation of lambic beer. Some human proteins also contain a PA14 domain, such as N-acetylgalactosamine transferase and fibrocystin.

As is the case for many cell wall proteins, the N-terminal domain of the PA14-Flo proteins is N-glycosylated and O-glycosylated [31,32]. For N-Flo1p, it has been shown that this protein is expressed in *S. cerevisiae* in two populations: one with an apparent molecular mass of 36 kDa and one of 100 kDa [32]. Both populations contain both short Man_8-14_GlcNAc oligosaccharides (core type) and large Man_>50_GlcNAc N-glycans (hyperglycosylated type) but in different ratios, i.e., two oligomannoses and one hyperglycosylated structure for the 36-kDa population, and one oligomannose and two hyperglycosylated structures for the 100-kDa population [39]. Electrospray ionization-mass spectrometry also revealed that N-Flo1p contains three O-glycosylation sites [39].

The central domain of Flo proteins contains many tandem repeats, which are rich in serine and threonine [1]. These repeats are indicated as “Flocculin repeats” (Flocculin family, Pfam entry PF00624) in the Pfam database (Figure 1B). Serine and threonine are prone for O-glycosylation. Clustered O-linked oligosaccharides induce the peptide core to adopt a stiff and extended conformation [36]. Also, the proline residues that are present in these repeats may prevent the central domain from forming a compact domain [1]. These aspects indicate that these adhesins are attached to the cell wall and stick out to interact with the mannose chains of other cells. Along the sequence, many consensus sequences for N-glycosylation, i.e., Asn-Xaa-Thr/Ser (Xaa represents any amino sequence except proline) [38], are present [23]. A few “Flocculin type 3 repeats” (Flocculin_t3, PF13928) are present close to the C-terminus of Flo9p (Figure 1B). This repeat is also present in Lg-Flo1p, Flo5p, and Flo10p, and in a number of other *Saccharomyces* proteins [1], but not in Flo1p (Figure 1B).

**Figure 2:**
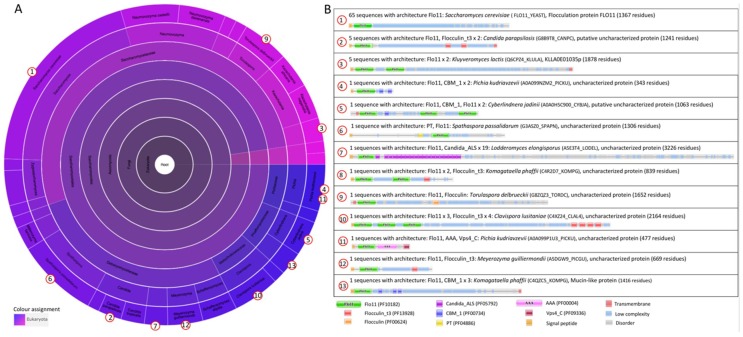
The family Flo11 (PF10182). (**A**) Sunburst phylogenetic representation of the Flo11 domain family. (**B**) Currently (July 2018), the Flo11 domain is present in 13 architectures and only within the ascomycetal orders of the *Saccharomycetales*. Indicated domains are Flo11 (PF10182); the “Flocculin type 3 repeat” (Flocculin_t3, PF13928) that is found in Flo9 close to its C-terminus and in a number of other *Saccharomyces* proteins [1]; the “Flocculin repeat” (Flocculin, PF00624) that is rich in serine and threonine residues [2]; “Candida agglutinin-like (ALS)” (Candida_ALS, PF05792) [3,4]; the “carbohydrate-binding module” (CBM_1, PF00734), which is found in carbohydrate-active enzymes [5,6]; the “PT repeat” (PT, PF04886), which is composed on the tetrapeptide XPTX; the ATPase family that is associated with various cellular activities (AAA, PF00004), in which AAA family proteins often perform chaperone-like functions that assist in the assembly, operation, or disassembly of protein complexes [7,8,9]; the “Vps4 C terminal oligomerization” domain (Vps4_C, PF09336) that is found at the C-terminal of ATPase proteins involved in vacuolar sorting, forms an α-helix structure, and is required for oligomerization [41]. The graphics were generated with Pfam version 31.0 [38].

**The Flo11 protein architecture.** The flocculin Flo11p is 37% similar to Flo1p (26% identical) [40]. Flo11p contains a Flo11 domain (family Flo11, Pfam entry PF10182) at its N-terminus (Figure 2B). The Flo11 domain is present in 13 architectures and only within the ascomycetal orders of the *Saccharomycetales* (Pfam version 31, [38]). The Flo11 domain is present at the N-terminal end. However, in some architectures, the Flo11 domain is present in double or triple copies, such as in *Klyuveromyces lactis* and the pathogenic yeast *Cl. lusitaniae* (Figure 2B). In some architectures, multiple “Flocculin type 3 repeats” (PF13928) are also present, such as in *Cl. lusitaniae* and *C. parapsilosis*. An uncharacterized adhesin of the pathogenic yeast *Lodderomyces elongisporus* [40], which is closely related to *C. parapsilosis*, contains multiple “Candida agglutinin-like (ALS)” (Candida_ALS, PF05792) domains close to the N-terminal Flo11 domain. For Flo11p from *S. cerevisiae*, the C-terminus is a GPI anchorage site [39] (although this is not present in the Pfam database (FLO11_YEAST) (Figure 2B)), which is also the case for some other adhesins.

#### 2.1.2. The Epithelial Adhesin Family

The adhesion of the opportunistic human pathogenic yeast *Candida glabrata* to epithelial cells is especially dependent on the interaction of the cell wall Epa (epithelial adhesins) proteins such as Epa1p, Epa6p, and Epa7p, which are the best characterized Epa adhesins [41,42,43,44,45,46]. The number of Epa members in *C. glabrata* depends on the strain: there are 17 in strain CBS138 and 23 in strain BG2 [47,48]. Epa-like adhesins are present in—besides *C. glabrata*—other species of the *Nakaseomyces* genus such as the human pathogenic *C. bracarensis* and *C. nivariensis*, which contain respectively 12 and nine *EPA*-like genes, and the non-pathogenic *Nakaseomyces delphensis* harbors a single copy, *C. castelli* contains three homologs of the EPA genes, and *N. bacillisporus* presented only one distant homolog [49]. These data indicate that the number of Epa-like adhesins is specifically enriched in pathogens, particularly in *C. glabrata*.

The N-terminal adhesion domain of Epa proteins contains a lectin activity, which is calcium-dependent and shows a certain similarity to domains within Flo1p and Agαp from *S. cerevisiae* [41,42]. Initially, this lectin domain was indicated as a PA14 domain (as for the PA14-Flo proteins) in the Pfam database, but is indicated now as the GLEYA domain (Pfam family GLEYA, PF10528). This domain is structurally related to the lectin-like binding domains that are found in the *S. cerevisiae* Flo proteins [42]. It is a carbohydrate-binding domain that is found in the fungal adhesins [50]. An EYDGA pentapeptide motif belonging to the PA14 domain was identified [24]. It is present in the N-terminal domain of Epa1 from *C. glabrata*, where it is involved in carbohydrate binding; and it is comparable to the VSWGT pentapeptide in Flo1p from *S. cerevisiae* [42]. The VSWGT motif of Flo1p and the EYDGA motif are present in the same position within a hypervariable region of the PA14 domain [24]. The VSWGT/KVLAR motif of Flo1p/Lg-Flo1p and the EYDGA motif of Epa1p represent a surface loop between two β-strands, 9 and 10, in the structure of the anthrax toxin PA domain [25]. Adhesins with a GLEYA domain possess a typical N-terminal signal peptide and a domain of conserved sequence repeats, but lack glycosylphosphatidylinositol (GPI) anchor attachment signals; the C-terminal location of their ligand-binding domains suggests an alternative form of cell wall attachment [44,46]. However, it was demonstrated for Epa1 that the GPI anchor is essential both for cross-linking in the cell wall and for Epa1-mediated adherence [45]. The GLEYA domain contains a conserved motif G(M/L)(E/A/N/Q)YA, hence the name GLEYA. Based on sequence homology, it is suggested that the GLEYA domain would predominantly contain β-sheets [51], which was confirmed by the solved structures of Epa1p and Epa9p (Table 1) [43,44].

The central domain of Epa proteins is rich in serine and threonine and has a structural function: it extends the N-terminal domain into the extracellular space outside of the cell wall mannoprotein layer, where it can interact with its ligand [45].

**Figure 3:**
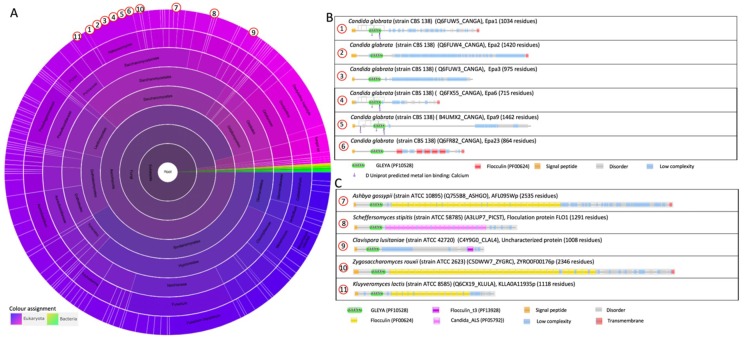
The family GLEYA (PF10528). (**A**) Sunburst phylogenetic representation of the GLEYA domain family. (**B**) Currently (July 2018), the GLEYA domain is present in 55 architectures and in 135 species, mostly belonging to the Fungi (97 species). Indicated domains are GLEYA (PF10528); “Flocculin type 3 repeat” (Flocculin_t3, PF13928) [1]; “Flocculin repeat” (Flocculin, PF00624) that is rich in serine and threonine residues [2]; “Candida agglutinin-like (ALS)” (Candida_ALS, PF05792) [3,4]. The graphics were generated with Pfam version 31.0 [38].

The GLEYA domain is present in 135 species and in 55 protein architectures (Figure 3A). A few examples of protein architectures containing the GLEYA domain at the N-terminus are shown in Figure 3C. The *Ashbya gossypii* AFL095Wp contains many Flocculin repeats (PF00624) in the central region. It has been shown that this gene *AFL095W* is a homolog of *S. cerevisiae FLO5* [52]. Similar architectures are shown for the ZYRO0F001p of *Zygosaccharomyces rouxii* and KLLA0A11935p of *Klyveromyces lactis*. A flocculation protein Flo1 is also present in *Scheffersomyces stipites*, where the central domain is composed of “Candida agglutinin-like (ALS)” (Candida_ALS, PF05792) domains. An uncharacterized protein of the pathogenic yeast *Clavispora lusitanae* contains 1 “Flocculin type 3 repeat” (Flocculin_t3, PF13928) at its C-terminal end.

#### 2.1.3. The Agglutinin-like Sequence Protein Family

The agglutinin-like sequence (Als) family includes eight members (Als1p-Als7p and Als9p) that share a high degree of sequence conservation [5]. The Als proteins from *C. albicans* have the typical three-domain modular design: an N-terminal region that contains a signal peptide and a conserved threonine-rich (T) region; a central domain with multiple tandem repeats; and a long, highly glycosylated Ser/Thr-rich C-terminal stalk region with a 13–20-residue signal sequence at the end for the attachment of a GPI anchor [5,53,54]. The N-terminus is relatively conserved among the Als proteins (55–90% similarity), poorly glycosylated, and extended to approximately 320–330 amino acids [5]. The ligand-binding domain is located within the N-terminus [55,56]. The binding domain is followed by a highly glycosylated T-domain that is rich in threonine residues [57]. It was shown that the T-region was necessary for N-Als folding, secretion to the medium, and cell wall anchorage [57]. The T-region has a conserved seven-residue sequence (IVIVATT) with amyloid-forming ability that is critical for cell aggregation and cell–substrate adhesion [58,59] (Figure 10).

The central region of the protein consists of a variable number of tandem repeats (TR) of about 36 amino acids in length, which play a critical role in the stabilization and proper presentation of the binding domain [55,57]. Based on molecular modeling and atomic force microscopy unfolding experiments, each tandem repeat folds into a discrete domain that can be unfolded [60]. The modeling data predicted a β-sheet-rich structure for individual repeats, which was experimentally confirmed by circular dichroism experiments [60]. The function of the TR region of Als5p was evaluated for the mediation of cell-to-cell aggregation and substrate binding: the aggregation was drastically reduced when N-Als5p was compared with the full-length protein [57]. The tandem repeats are highly hydrophobic, but the surrounding O-glycans are hydrophilic, resulting in an aggregation mediated through the hydrophobic effect [60]. The tandem repeats also mediated adherence to fibronectin and polystyrene [57].

The C-terminus is the least conserved in length and sequence among the Als proteins, and is extensively glycosylated due to a large number of serine and threonine residues [60]. This part of the protein is also referred to as a stalk through which the N-terminal binding domain extends away from the cell surface, and can interact with its ligand. The C-terminus contains a GPI anchor sequence that interfaces with the cell wall [5].

**Figure 4:**
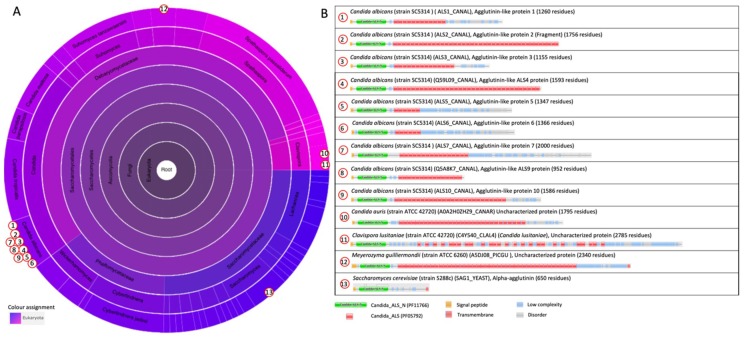
The domain family “Cell-wall agglutinin N-terminal ligand-sugar-binding” (Candida_ALS_N PF11766) [42]. (**A**) Sunburst phylogenetic representation of the “Candida_ALS_N” domain family. (**B**) Currently (October 2018), the Candida_ALS_N domain is present in 38 architectures and only within the ascomycetal orders of the *Saccharomycetales*. Indicated domains are Candida_ALS_N (PF11766), which is likely to be the sugar or ligand-binding domain of the yeast alpha agglutinins [42]; “Candida agglutinin-like (ALS)” (Candida_ALS, PF05792) [42,51]. The graphics were generated with Pfam version 31.0 [38].

The N-terminal binding domain is classified as the “cell-wall agglutinin N-terminal ligand–sugar-binding” domain family (Candida_ALS_N, PF11766) in the Pfam database. The Candida_ALS_N domain is present in 38 architectures, and only within the ascomycetal orders of the *Saccharomycetales* (Figure 4A). The architectures of proteins that contain Candida_ALS_N, such as the Als protein family, are illustrated in Figure 4B. These Als proteins contain repeated “Candida agglutinin-like ALS” (Candida_ALS, PF05792) domains in their central region, and for Als2p and Als4p, also up to the C-terminus. This Candida_ALS domain corresponds to the previously described tandem repeats. Other examples of architectures shown in Figure 4B are the *S. cerevisiae* α-agglutinin, and uncharacterized proteins from *Candida auris*, *Clavispora lusitaniae*, and *Meyrozyma guilliermondii* that both contain repeated Candida_ALS domains in their central region. *C. auris* is an emerging multidrug-resistant pathogen that causes invasive infections, particularly among hospitalized patients with significant medical comorbidities [45,46,47,48,49,61]. It was first described in 2009 in Japan [50], and has been reported from several countries since. *C. auris* contains an uncharacterized adhesin with the same architecture as that of the Als adhesins (Figure 4B).

### 2.2. Protein Structures of Yeast Adhesins

#### 2.2.1. The PA14 Fold in Flocculation Protein and Epithelial Adhesin Family

The first structure of the flocculation proteins that was solved was the N-terminal adhesion domain N-Flo5p [53] (Table 1). Next, the structures of the *C. glabrata* adhesins Epa1p [43,44] and later also Epa6p and N-Epa9p were solved [52] (Table 1). N-Lg-Flo1p from *S. pastorianus* had been crystallized and diffracted to high-resolution using X-ray radiation [65], but due to a phase problem, it could only be solved when the structure of N-Flo5p was known, since it was used as a search model by molecular replacement [54]. Next, the N-Lg-Flo1p and N-Flo1p in complex with their ligands were solved [39] (Table 1).

**Figure 5:**
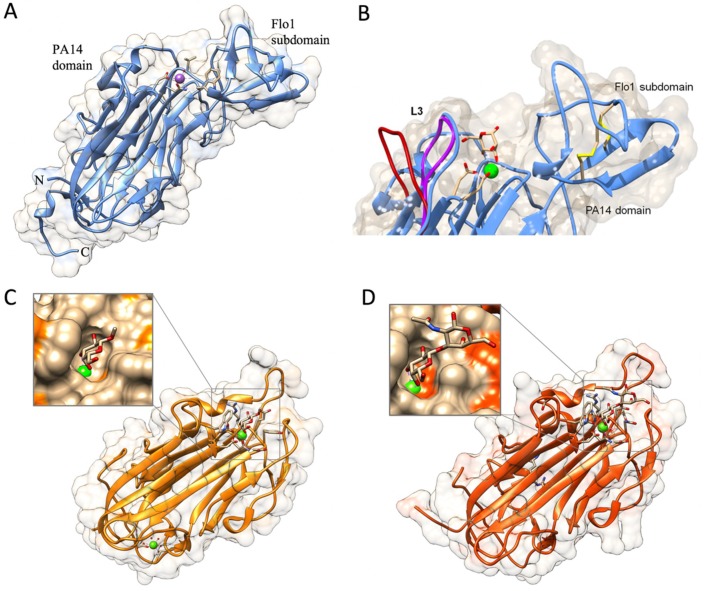
(**A**) N-Flo1p (**B**) Partial view of the N-Flo1p binding pocket (light blue ribbon/surface) in complex with calcium–mannose. Conformations of the L3 loop from N-Lg-Flo1p (purple) and N-Flo5p (crimson; from PDB entry 2XJP) bound states are also shown for comparison. Reprinted from [39]. (**C**) Overall fold of N-Epa1p with zoomed view of the interaction of galactose to Ca^2+^ in the binding pocket (PDB entry 4A3X). (**D**) N-Epa6p with zoomed view of the binding of the T-antigen in the binding pocket (PDB entry 4COW).

The overall atomic structures of N-Flo5p, N-Flo1p, N-Lg-Flo1p, N-Epa1p, N-Epa6p, and N-Epa9p are very similar. These structures confirmed the topological link between the flocculins and the PA14 domain [39,53,54]. The main body of these proteins, i.e., the PA14 domain, is a β-sandwich fold made up of two antiparallel β-sheets and an L-shaped region composed of the N and C-terminal regions (Figure 5A,B). N-Flo1p and N-Flo5p contain a protruding β-sheet subdomain (the Flo1/Flo5 subdomain) that is located at one end of the protein, close to the carbohydrate binding site (Figure 5A,B). These subdomains are stabilized by two disulfide bonds. In Lg-Flo1p and N-Epa1, this subdomain is replaced by a short highly flexible loop 2 (L2) [39,54]. The high flexible loop 3 (L3) is present in N-Flo1p (Fig. 5B), N-Flo5p, and N-Lg-Flo1, as well as in N-Epa1 [43,44]. This loop plays a significant role in carbohydrate recognition. In N-Flo1p, this loop is closer to the binding side (Fig. 5B), and lysine 194 (K194) from this loop can directly interact with the carbohydrate, in contrast to N-Flo5p. This results in a three-fold increased affinity for mannose in N-Flo1p compared to N-Flo5p. In Epa1p, the L3 loop via tryptophan 194 (W194) (which corresponds to K194 in Flo1p) establishes stronger stacking interactions with the ligands galactose and galactose-terminating glycans [43,44]. The binding site of these proteins contains a calcium ion that is directly involved in carbohydrate binding (Figure 5). In N-Flo1p and N-Flo5p, Ca^2+^ is coordinated on carbohydrate binding loop 1 (CBL1) by *cis* peptides aspartic acid 160 (D160) and D161 (indicated as “D*cis*D” motif), and on CBL2 by the asparagine 224 (N224) side chain and the carbonyl groups of valine 226 (V226) and W228. Residues D160, D161, and N224 are strongly conserved in the Flo and Epa adhesin families due to their importance for metal binding [44,53]. The N-Lg-Flo1p carbohydrate-binding pocket is more enclosed than the one of N-Flo1p, which results in a much higher affinity for mannose (Table 2) [39]. There is a distinct variation in the ways that mannose disaccharides and high-mannose glycans fit in the binding sites of N-Flo1p and N-Flo5p, which results in a different specificity and affinity for these carbohydrates (Table 2). Longer mannose-containing oligosaccharides do not interact well with N-Lg-Flo1p due to the steric hindrance encountered in the binding site (Table 2).

Recently, a large-scale functional analysis of the N-terminal adhesion domains of 17 Epa paralogs in combination with three-dimensional structural studies of N-Epa1p and Epa6p with cognate ligands was performed, and revealed that most Epa paralogs possess individually tailored ligand-binding properties [52]. Most Epa adhesin domains exert lectin-like functions, and together recognize a wide variety of glycans with terminal galactosides. It was shown that the Epa adhesin domains of functionally closely related members (such as N-Epa6p and N-Epa13p or N-Epa1p and N-Epa3p) are structurally quite diverse; and *vice versa*, phylogenetically closely related adhesins (such as N-Epa6p and N-Epa7p or N-Epa3p and N-Epa3p) possess distinct ligand-binding affinities, which indicates that functionally related Epa variants might have repeatedly developed independently. A comparison of the variability/conservation of amino acid residues located on the protein surfaces of the adhesin domains of the different Epa members or their ligand-binding pockets revealed that all of the Epa adhesin domains have conserved PA14/GLEYA-like cores and a highly variable surface composition. Two signatures, i.e., the “DD-N” and “W-R” signatures, constitute an invariable core of the binding pocket that are essential for the efficient binding of the terminal hexose moiety in most Epa adhesin domains [52]. The DD-R signature refers to the D*cis*D motif of CBL1 and an asparagine of CBL2 that confer coordination of the Ca^2+^ ion (see above). The W-R signature refers to a highly conserved tryptophan from loop L3 at the surface, and an arginine from a corner of the inner binding pocket (CBL2) [44]. The Epa adhesin domains contain three highly variable residues within the CBL2 region that contribute to the ligand specificities of the different Epa members [44,52]. Saturation mutagenesis based on the structure of N-Epa1p and the role of the loop CBL2 resulted in two mutants (E227A) and Y228W) with improved binding affinities for fibronectin [55]. Glycan array screening also revealed that single-point mutations in CBL2 changed the carbohydrate specificity toward sulfated glycans.

#### 2.2.2. The Flo11 Fold

Planktonic *S. cerevisiae* cells can switch to complex multicellular structures such as flocs, filaments, mats, and flors [6,55]. The flocculation protein Flo11p has a major role in these lifestyles [40,56,57,58]. Upon glucose depletion, *FLO11* (previously also indicated as *MUC1*) gene expression renders haploid cells adherent and invasive into semi-solid agar medium (called “invasive growth”) [8,59,60]. In diploid pseudohyphal growth, the cells adopt an elongated shape and form filaments that grow from the colony edge under low nitrogen concentration conditions [60,66,67]. *FLO11* expression is also associated with the formation of mats, which are complex colony-like structures on a low-density semi-solid medium [68,69]; the formation of a flor (or velum), which is the air-liquid interfacial cellular aggregation in the process of sherry-like wine fermentations [65,70,71]; the adherence of cells to a range of solid surfaces (such as glass, stainless steel, agar, and plastics) can also lead to the development of biofilms [60,68,72]. The expression of *FLO11* is also involved in cell–cell interaction (floc formation) [40,60,64,73,74,75,76]. Many parameters influence the expression of *FLO11* and flocculation activity such as the cell density, surface charge, and pH, and environmental factors such as oxygen limitation, nutrient limitation, and cell surface hydrophobicity [73,77,78,79]. It was shown that Flo11p mediates different processes in different strains [8,29,40,57,60,68,69,73,74,75]. Experimental data indicate that strain-specific differences in the level of flocculation result from significant sequence differences in the *FLO11* alleles, and do not depend on quantitative differences in *FLO11* expression or surface hydrophobicity [80].

**Figure 6:**
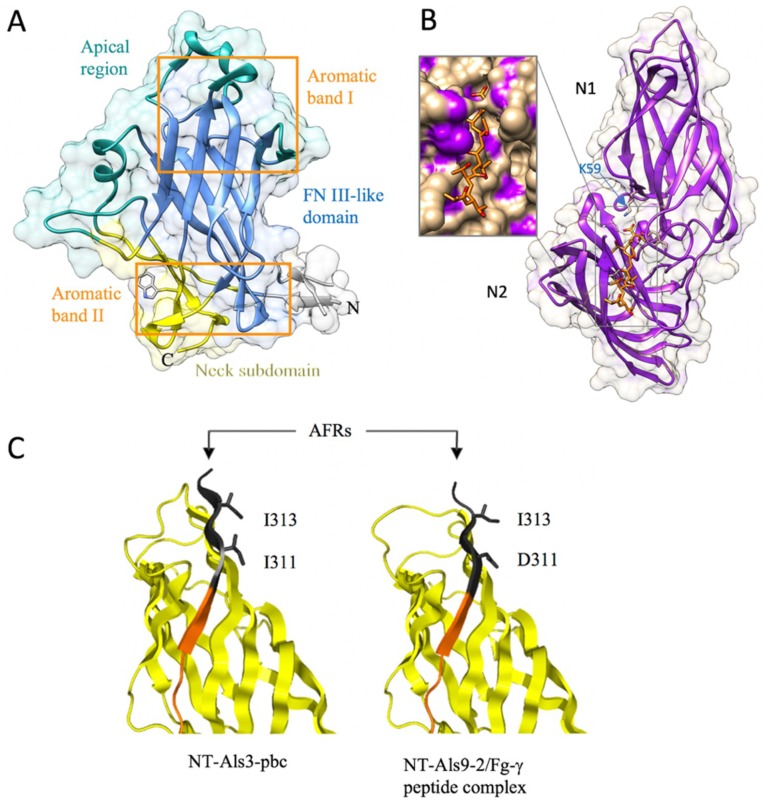
(**A**) The Flo11 adhesin (N-Flo11p) is composed of three domains: the apical region, the fibronectin type III domain (FN3), and the neck subdomain (4UYR). Various Trp and Tyr are present around the ends of the elongated protein domain, and these two regions are indicated as aromatic band I and II. (**B**) N-Als9-2p with bound peptide (heptathreonine) (4LEB) with an indication of the two Ig domains N1 and N2 and lysine (K59) that is involved with the binding of the carboxylate end of the peptide ligand. (**C**) N-Als3p and N-Als9-2p with indication of the position of the amino acids substituted to abolish the amyloid-forming region (AFR) function. The side chains of residues Ile-311 and Ile-313 were exposed to solvent in the structure of NT-Als3-pbc (left), which recreated the conformation of the ligand-bound form of the protein, as shown in the structure of NT-Als9-2p in complex with an Fg-γ peptide (right). For comparison, the equivalent residues in N-Als9-2p (Asp-311 and Ile-313) are shown. Substitution of Ser for each Ile residue in N-Als3p eliminated the amyloidogenic propensity of the AFR. Reprinted with permission from [62].

The structure of the N-terminal adhesion domain of the Flo11 adhesin has been recently solved using X-ray crystallography [64] (Table 1). N-Flo11p is composed of three subdomains: a hydrophobic apical region, a β sandwich of the fibronectin type III domain (FN3-like domain), and the neck subdomain (Figure 6A). The core domain is the β sandwich that is formed by the antiparallel β sheets I and II, and was assigned to the class of fibronectin type-III like domains (FN3). The FN3 fold forms a large family within the immunoglobulin (Ig) superfamily that includes cell adhesion proteins, cell surface hormone and cytokine receptors, chaperones, and carbohydrate-binding domains [81]. The FN3-like domain subtype shows a seven-stranded strand-switched type, with sheet I consisting of three strands and sheet II of four strands (Figure 6A). The FN3 fold differs from other Ig folds by its fourth strand, which is part of the second, but not the first, β sheet [64].

The FN3-like domain is girdled by two surface aromatic bands at the apical region and the neck subdomain [64]. Hydrophobic interactions between these aromatic surface features, whose propensity for interaction is ameliorated in a pH-dependent manner by co-distributed acidic residues, mostly determine the homophilic recognition by the Flo11 adhesin domains (Figure 10). Although these hydrophobic interactions are less specific than the lectin–carbohydrate interactions of the other Flo adhesins, they can excel by their long range of attractive forces (100–200Å). The co-alignment of Flo11 fibers from opposing yeast cells could be observed by scanning electron microscopy, indicating that Flo11p acts as a spacer-like, pH-sensitive adhesin that resembles a membrane-tethered hydrophobin [64].

#### 2.2.3. The Ig-like Fold in Agglutinin-like Sequence Protein (Als) Family

The first structure of the Als proteins that was determined was the N-terminal domain of Als9 (N-Als9-2p) (18–329 amino acids) [82,83] (Table 1). Nuclear magnetic resonance (NMR) data revealed an IgG-superfamily secondary-structure topology [82]. Using X-ray crystallography, it was revealed that N-Als9-2p contains two immunoglobulin-like (Ig) domains (N1 and N2) that present a general MSCRAMM-like (microbial surface component recognizing adhesive matrix molecules) fold [83] (Figure 6B), which is typical for the fibrinogen-binding adhesin SdrG from *Staphylococcus epidermis* [84,85] and ClfA from *S. aureus* [86]. The peptide from the C-terminal end of human fibrinogen γ (Fg-γ) binds in a deep-binding cavity formed by two β strands from one Ig domain and a loop from the second domain. This binding cavity is limited to contain up to six residues of the ligand, and ligand recognition relies on a motif that is capable of binding the flexible C-termini of peptides in extended conformation. Central to this mechanism is an invariant lysine residue (K59) at the end of the binding cavity that recognizes the C-terminal carboxylate of peptide ligands (Figure 6B), which allows the remaining peptide backbone to associate in parallel orientation with β-strand G2 [83].

The N-terminal adhesion domain of Als proteins, in particularly Als3p [87], can bind to numerous ligands [83,88,89,90,91,92,93]. To determine the molecular mechanism of interaction, mutations in N-Als3p that disrupt the peptide-binding cavity (PBC) function were designed based on the N-Als9-2p structure [62]. The loss of PBC function resulted in an adhesive phenotype that was indistinguishable from the *als3* deletion strain. The N-Als C-terminus that contains a conservative amyloid-forming region (AFR), which also contains adhesive properties [91,93], was also targeted [62]. *C. albicans* with destroyed Als3p amyloidogenic potential showed little contribution of the AFR to adhesion, and suggested an aggregative function of the AFR.

Als3p and Hwp1 (hyphal wall protein 1) are involved in the binding of *C. albicans* hyphae to epithelial cells [94,95,96,97,98,99,100]. Hwp1 is related to the Hwp2p and Rbt1p proteins that belong to the Hwp1 family [2], since they share a highly conserved 42-aa repeat unit, although their N-terminal effector domains do not have sequence similarities [101,102]. These Hwp family proteins are required for adhesion to host cell surface proteins, cell-cell aggregation, mating, and biofilm formation [103,104,105,106,107,108]. Hwp1p has a N-terminus that is highly enriched in glutamine residues, which are substrates for human host transglutaminase enzymes [94,96,109]. These enzymes covalently cross-link Hwp1p to the ECM proteins of epithelial cells.

## 3. Yeast-Yeast and Yeast-Host Cell Interactions

### 3.1. Flo Proteins Interactions

#### 3.1.1. Cell-Cell Binding Based on *S. cerevisiae*-Lectin-Flocculin Interaction

PA14-lectin flocculins recognize the disaccharidic ends of glycans. N-Flo1p interacts specifically with D-mannose glycans [110], which is an interaction that is characterized by a millimolar affinity [39] (Table 2). The affinity of N-Flo5p for D-mannose is three times lower [53]. The affinity of N-Flo1p and N-Flo5p is around 10 times larger for disaccharides than for monosaccharides. N-Flo1p and N-Flo5p bind stronger to α-1,2-mannobiose than mannose (Table 2). The adhesion domain of Flo5p binds to mannose-α1,2-mannose disaccharides with 10 times higher affinity than to mannose. N-Flo1p binds to α-1,3-linked and α-1,6-linked mannobiose saccharides, whereas N-Flo5p does not. N-Flo1p and N-Flo5p interact weakly with glucose. N-Lg-Flo1p displays a broad specificity toward sugars and has a 14-fold higher affinity for mannose-1-phosphate and glucose-1-phosphate compared to their unphosphorylated counterparts (Table 2) [54]. N-Lg-Flo1p has a micromolar affinity for glucose-1-phosphate and a millimolar affinity for glucose (Table 2). The interaction of Lg-Flo1p that is present on the surface of a lager yeast with glucose could be determined quantitatively by determining a rupture force of 121 pN using AFM single-molecule force spectroscopy (Table 3). Its interaction with D-mannose was characterized by a micromolar affinity, whereas for mannobioses, it was characterized by a millimolar affinity.

N-Flo1p also binds to N-Flo1p; this homophilic interaction has millimolar affinity [39] (Table 2). AFM single-molecule force spectroscopy confirmed this interaction: a rupture force of around 300 pN was determined [39] (Table 3).

**Figure 7:**
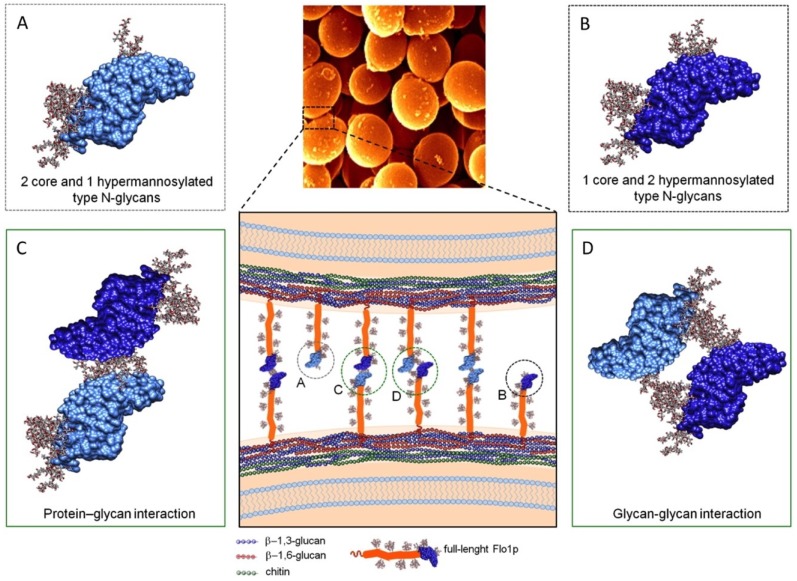
Molecular flocculation model based on Flo1p homophilic self-interaction. The two populations of N-Flo1p, which carry different types of N-glycans, are illustrated. Two *S. cerevisiae* cells bind together via Flo1p self-interaction. This binding is accomplished via lectin-glycan and glycan-glycan interactions. (**A**) 36 kDa of N-Flo1p containing two short core type N-glycans (Man_8-14_GlcNAc oligosaccharides) and one large hyperglycosylated type N-glycans (Man_>50_GlcNAc). (**B**) 100 kDa of N-Flo1p containing one short core type and two large hypermannosylated type N-glycans. (**C**) Lectin–protein interaction. (**D**) Glycan-glycan interaction. Reprinted from [39].

N-Flo1p is expressed as two populations with different molecular masses of 36 kDa and 100 kDa [110] and is both O and N-glycosylated at three sites [39] (Figure 7). In Flo1p-Flo1p interactions, N-Flo1p self-interacts homophilically with the glycans of N-Flo1p in the presence of Ca^2+^. On flocculating cells, Flo proteins are the dominating cell wall proteins that stick out of the cell wall [113], and therefore, N-Flo1p interactions will almost exclusively be responsible for the flocculation phenotype. The low affinity of N-Flo1p self-interaction and binding to mannose could guarantee that the occasional binding of Flo1 proteins on the same cell is abolished quickly, and allows Flo1p to subsequently interact with a Flo1 protein on a different cell [39]. Another hypothesis stated that the binding of Flo5 proteins on the same cell is prevented due to the presence of a second binding site, since one binding site could then form *cis* interactions that immobilize the N-terminal domain at the cell surface, while the other binding site is responsible for the *trans* interaction [53]. Besides Flo1p-mannose interaction, it was also shown that glycan-glycan interactions contribute to cell-cell interaction [39], and these interactions are likely involved in the first intercellular contacts [114,115,116,117]. It was demonstrated that Ca^2+^ is also involved in N-Flo1p glycan-glycan interactions. This points to a two-step cell-cell adhesion process, where in a first step the long, flexible glycans have a high probability of interaction when cells are moving close to each other and initially serve to stabilize cell-cell interactions. In a next step, the non-reducing glycan ends enter the binding pocket of the Flo lectin, and binds to the protein. In both steps, Ca^2+^ plays a crucial role in the interactions. This flocculation model unifies the historically first-proposed model “Ca^2+^-bridge” hypothesis [118,119] with the generally accepted lectin hypothesis [120]. The “Ca^2+^-bridge” hypothesis stated that flocculation is based on ionic interactions stabilized by hydrogen bonds and the involvement of Ca^2+^ ions that could form bridges between flocculating cells by linking the carboxyl groups present on the cell surface [118]. In the two-step model, Ca^2+^ could bridge cells through glycan-glycan interactions via negatively charged phosphates that are present in hyperglycosylated N-glycans (Figure 7D).

#### 3.1.2. Cell-Cell Binding Based on *S. cerevisiae*-Flo11 Protein Interaction

**Figure 8:**
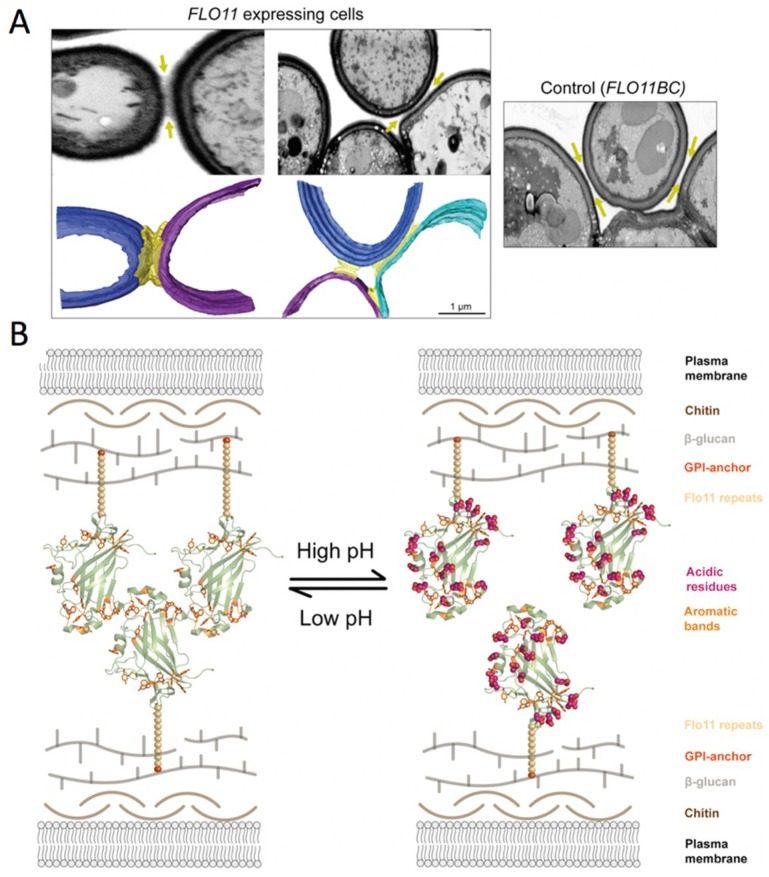
(**A**) Ultrastructural view and model of cell-cell contact sites with spacing as provided by Flo11p-Flo11p interactions. The electron microscopy photographs depict the filamentous nature of the cell-cell contacts (arrows). Three-dimensional (3D) reconstructions of the cell walls from different *FLO11*-expressing cells are shown in blue, cyan, and purple, while the Flo11p-Flo11p interaction layer is shown in yellow. The control cells lacking the Flo11 adhesin domain (right) demonstrate the lack of intercellular filamentous structures at the cell-cell contacts (arrow). (**B**) Model of Flo11p–Flo11p interactions where N-Flo11p–N-Flo11p cluster in an oriented way by the interaction of the aromatic bands, which is pH-dependent. Reprinted with permission from [64].

*S. cerevisiae* cells that express *FLO11* interact via N-Flo11p-N-Flo1p interactions (Figure 8) [64,76]. Recent data show that Flo11p acts as a spacer-like, pH-sensitive adhesin that resembles a membrane-tethered hydrophobin [64]. This homophilic N-Flo11p-N-Flo11p interaction depends mostly on hydrophobic interactions. These interactions are mediated by the two aromatic bands that are present at the ends of the adhesion domain (Figure 6A and Figure 8B). The tryptophan and tyrosine residues forming the aromatic bands are well-conserved in the Flo11 protein family. These bands on the surface are lined by stretches of acidic residues (Figure 8B), which determine the pH sensitivity of the adhesive functions, i.e., cell-cell interaction and adhesion to hydrophobic plastic surfaces [72]. Homophilic N-Flo11p is pH sensitive: interactions could only be observed using acidic buffers close to the isoelectric point of N-Flo11p (Figure 8B) [64]. Electron microscopy imaging revealed the ultrastructure of the cell-cell contacts: a significant space of 100–200 nm filled with filamentous material (Figure 8A) [64,125]. The Flo11-dependent fibers between cells co-align upon close contact, but the overall structure is highly unordered [64].

### 3.2. C. albicans Als Protein Interactions

Als1p and Als3p are the best studied Als proteins. The N-terminal domains of both proteins interact with a broad range of ligands, such as fibronectin, laminin, collagen IV, fibrinogen, and gelatin [125,126,127,128]. N-Als1p and N-Als3p have a 10-times higher affinity for laminin than for fibronectin (Table 2). It was also shown that N-Als1p has a lectin-like activity, since a sub-millimolar affinity toward fucose-containing glycans, and preferentially with antigen H type 2 that are present in blood group antigens, was detected [111]. N-Als3p also interacts with carbohydrates such as long chains of repeated LacNAc (Galβ-1,4-GlcNAc); the micromolar affinity for GlcNAc was determined (Table 2). GlcNAc is part of type 1 LacNAc (Galβ-1,3-GlcNAc) and type 2 LacNAc (Galβ-1,4-GlcNAc) structures that build the scaffold for blood group H and Lewis-type units [5,129].

**Figure 9:**
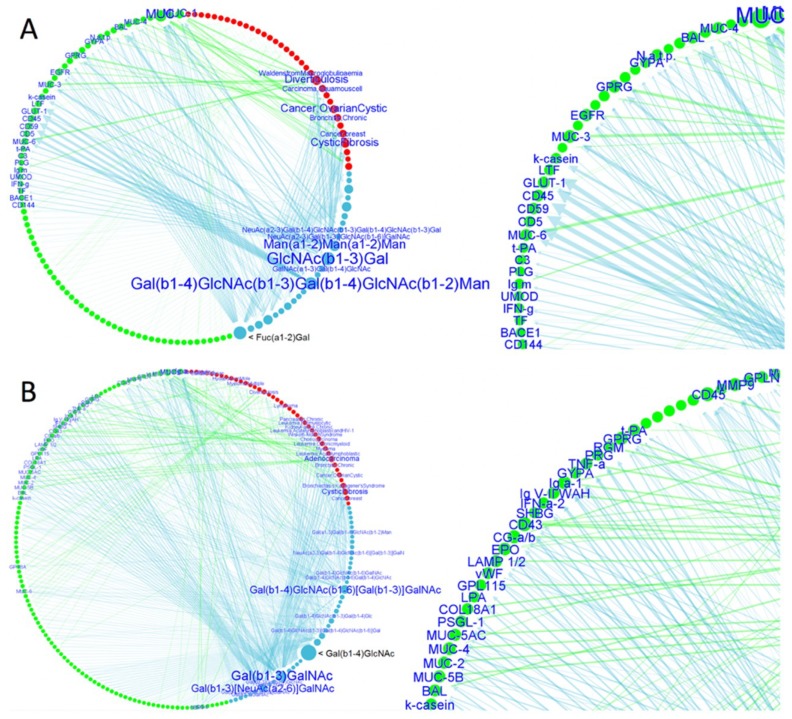
Lectin-glycan interaction networks (LGI) of agglutinin-like sequence (Als) and epithelial adhesins (Epa) proteins. Glycan determinant data and their connections with human glycoproteins and related diseases are depicted for *C. albicans* Als proteins (N-Als1p and N-Als3p) (**A**) and Epa proteins (N-Epa1p, N-Epa5p, and N-Epa7p) (**B**). Reprinted with permission from [112]. Close-up views of the networks are shown on the right. The nodes’ dimensions and the arrow thickness/label size depend on the number of connections and the glycan-binding strength, respectively. Notably, the determinants Fuc(α1-2)Gal (**A**) and Gal(β1-4)GlcNAc (**B**) are both characterized by a high number of connections (large node, i.e., several human glycoproteins are characterized by the presence of these glycan determinants), but a low relevance. No label is shown; i.e., the Epa/Als intensities of binding to the glycans that contain these determinants are lower than the other determinants. Reprinted from [112].

Microbial adhesion to components of the glycocalyx, such as glycosylated host receptors or other glycoproteins, is in many cases mediated by adhesins endowed with a lectin activity [4]. Since Als1p and Als3p could be classified as lectin-like adhesins, a lectin–glycan interaction (LGI) network can be constructed [112] (Figure 9 A). This approach is based on linking the glycan array screening data of lectin-like adhesins to a human glycoprotein database via the construction of an LGI network, and can be used to profile potential adhesin binding receptors in the host with prioritization of the most relevant interactions. This network reveals a large set of potential human binding receptors for Als adhesins. Several glycan determinants are linked to mucins, which are the main constituents of the extracellular secreted mucus and GalNAc-rich cell surface glycocalyx. Previously, it was demonstrated that there was a link between *C. albicans* adhesion to human cells and mucin [130,131]. The affinity of N-Als3p for GlcNAc in the network was confirmed by the determination of the dissociation constant (Table 2).

**Figure 10:**
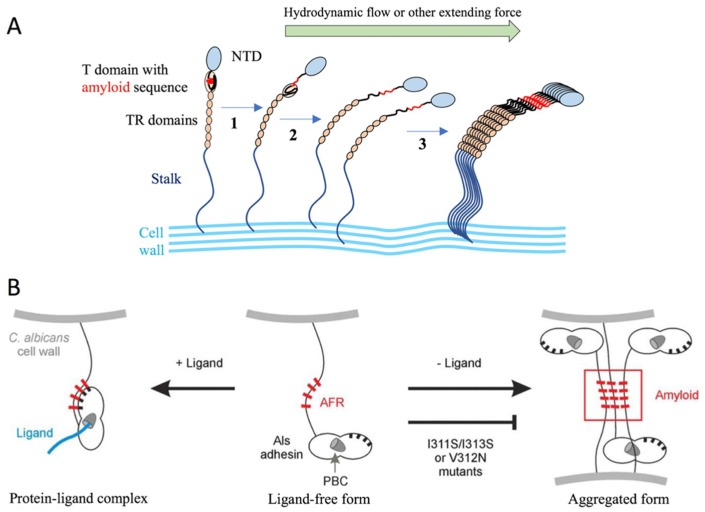
Models proposed that explain the function of the amyloid-forming region (AFR) in Als protein interactions. (**A**) Force-induced aggregation of Als proteins on the surface of the same cell. In the initial state, the amyloid core peptide is buried in the interface between the N-terminal adhesin domain (NTD) (blue) and the T-domain. In step 1, shear stress unpacks this interface; in step 2, the T-domain unfolds, allowing flexibility to promote interactions among the adhesins to form a nanodomain (step 3). Adapted from [134]. (**B**) Proposed conformations of the AFR in Als adhesins. Newly synthesized Als3 in “free form” (center) is competent for ligand binding via the peptide-binding cavity (PBC) or for aggregation mediated by the AFR. Interaction between the AFR of Als3 proteins on different *C. albicans* cells leads to the formation of aggregates (amyloid; right). Mutations in this region (e.g., V312N [132] or I311S/I313S [62]) abolish this phenotype. In the presence of ligands, the AFR attaches to the surface of the adhesin (left) [83]. High-affinity ligands are predicted to shift the equilibrium toward this non-aggregative protein-ligand complex. If aggregative interactions are disrupted by mutation of the AFR, the PBC could become more available to bind ligands. Reprinted from [62].

It has been shown that the strength of Als-mediated adhesion is partly the result of the force-activated amyloid-like clustering of hundreds of adhesins to form arrays of ordered multimeric binding sites (Figure 10A) [132,133,134]. Single-molecule AFM experiments demonstrated that two-dimensional Als5 protein clusters could be observed on the cell surface following the application of extension force to single molecules by the AFM tip [92]. The clustering is a result of surface amyloid formation, and depends on specific amino acid sequences with extremely high β-aggregation potential. A peptide containing the high-potential amyloid core sequence binds specifically to the surface of cells with nanodomains. This leads to the formation of surface nanodomains and adhesion. It has been shown that these properties apply also for other *C. albicans* adhesins and *S. cerevisiae* Flo1p and Flo11p [134]. The clustering is facilitated by the length and flexibility of the unstructured stalk region of the adhesins. Another model stated that newly synthesized Als proteins can either bind ligands via the peptide-binding cavity (PBC), which results in attaching the amyloid-forming region (AFR) to the N-terminal domain surface or using the free AFR to interact with other AFRs, which forms protein and cellular aggregates [62,135] (Figure 10B).

*C. albicans* adhere to both biotic and abiotic surfaces, and can result in biofilm formation. These biofilms are a significant medical problem, because they commonly form on implanted medical devices, are drug-resistant, and are difficult to remove [136,137,138,139,140,141]. This feature together with the ability to adhere to other *C. albicans* cells contributes to the structural integrity of biofilms and is the first step in biofilm formation [142]. The nature of the surface, molecules involved in quorum sensing, host hormones, and the presence of other interacting microorganisms can influence the initial step of biofilm formation [83,142,143,144,145,146,147,148,149]. Time-dependent gene expression analysis during biofilm development revealed that genes involved in both adhesion and metabolism are at the core of biofilm development [141].

### 3.3. C. glabrata Epa Protein Interactions

Most Epa adhesion domains exert lectin-like functions and together recognize a wide variety of glycans with terminal galactose linked via α or β-glycosidic bonds to a secondary sugar for conferring epithelial cell adhesion [52]. Phylogenetically closely related adhesins, such as Epa6p and Epa13p, or Epa3p and Epa22p, possess markedly distinct ligand-binding specificities. N-Epa1p, N-Epa6p, and N-Epa7p confer the most efficient epithelial binding [52]. Epa1p and Epa7p bind galactose-containing glycans with a specificity for β-1,3- and β-1,4-linked galactose moieties, but they show a preference for glycan structures containing the core 1 structure of mucin-type O-glycans, also named the T antigen (Galβ-1,3-GalNAc) [44,150]. In contrast, Epa6p is not able to discriminate between α and β-glycosidic linkages. N-Epa1p and N-Epa7p prefer Gal and GalNAc over GlcNAc as the second hexose moiety at the end of the glycan (Table 2). N-Epa6p is almost unspecific for the discrimination between different glycosidic linkages and significantly less specific for discriminating between Gal, GalNAc, and GlcNAc at the secondary position of the disaccharide (Table 2) [52]. These Epa adhesins bind these carbohydrate ligands with micromolar affinity (Table 2). Single-cell AFM force spectroscopy revealed that the force to unbind a single *C. glabrata* cell that was adhered to a hydrophobic surface was in the range of 30 nN to 50 nN (Table 3).

Since most Epa proteins are lectin-like proteins, an LGI network was constructed based on glycan array data for N-Epa1p, N-Epa5p, and N-Epa7p (Figure 9B) [112]. As for Als proteins, several glycan determinants are linked to mucins. The binding of Epa1p, N-Epa6p, and Epa7p to mucin-type O-glycans had been described, i.e., the affinity for the T-antigen, which constitutes the core 1 structure of mucin-type O-glycans [43,44,150]. The three Epa proteins are linked in the network to the mucins that carry the T-antigen and/or the sialyl-T antigen and are associated with diseased states, i.e., colon adenocarcinoma (MUC1, MUC2, MUC4, MUC5A/B/C), breast and uterine cancers (MUC1), and lung diseases, which may cause bronchiectasis (MUC). The interaction of N-Epa1p for mucin was confirmed by determining the dissociation constant (Table 2). Another interaction that was revealed in the network is the interaction with fibronectin (Figure 9B), i.e., Epa1p and Epa7p are linked to the fibronectin of fibroblasts by LacNAc-terminated N-glycan branches. Binding inhibition experiments could also confirm that the observed interactions with fibronectin and mucin are mediated by galactose-containing glycans that are attached to fibronectin and mucin [112].

## 4. Conclusions

As discussed in this review, only three-dimensional (3D) structures of a few adhesins belonging to three protein families of the yeast species *S. cerevisiae*, *C. albicans* and *C. glabrata*, have been solved. Additionally, only the structures of the N-terminal adhesion domains of these adhesins were solved; no full-length structure was known until today. Pfam database mining shows that the structural domains that are present in these solved structures are also present in many other fungi (and bacteria), including many pathogenic yeasts, and they appear in various protein architectures. Three-dimensional structural determination of fungal adhesins using classical X-ray diffraction is still difficult, since they are large and highly glycosylated. Nuclear magnetic resonance (NMR) spectroscopy is performed in solution, and can help with understanding the binding mechanisms of ligands, as was demonstrated for N-Als9-2p [83]. Future structural biology research of adhesins will certainly be based on using modern cryo-electron microscopy (EM) methods, since cryo-EM has recently evolved toward a near atomic resolution structure determination of proteins in native conditions [151]. For example, the type-1 chaperone-usher pilus rod structure of uropathogenic *Escherichia coli* was recently determined at 4.2-Å resolution using cryo-EM [152].

Structural studies have to be complemented with biophysical interaction studies at the molecular and cellular level to determine the adhesion mechanism. In the future, fungal adhesion data in structural and functional databases will be more and more linked and used to unravel the complex interactions of various microbial (fungal, bacterial, and viral) pathogens that are involved in many infectious diseases. Recently, a database strategy, i.e., the lectin–glycan interaction network strategy, was set up to predict interacting host receptors for the *Candida* adhesins Als and Epa, and the bacterial uroepithelial FimH adhesin from *E. coli* [112]. In this strategy, a database of experimental lectin-binding data obtained by glycan array screening was linked to a glycoproteomic database. Since the glycan structure of these proteins can be modified in disease states, a link between adhesin interaction and some diseases could be established.

## Figures and Tables

**Table 1:** Protein structures of yeast adhesins deposited in the Protein Data Bank (PDB, www.rcsb.org).

Micro-Organism	Adhesin	Ligand in the Structure	Mutations	PDB Code	Interacting Substrate/Function Properties	Refs
*C. albicans*	NT-Als3p (residues1-313)	Heptathreonine	-	4LEB	Endothelial and epithelial cells; fibronectin, laminin, type-IV collagen; abiotic surfaces (glass, plastics)	[62]
		Apo	K59M, A116V, Y301F	4LEE		[62]
	NT-Als3p (residues 1-299)	Apo	-	4LE8		*
	NT-Als9-2p	Apo	-	2Y7N		[63]
		Apo	G299W	2Y7O, 2YLH		
		Fg-γ ^1^	-	2Y7L		
*C. glabrata*	N-Epa1p	Gal	-	4A3X	Epithelial cells, fibronectin, mucin	[43]
		Galβ-1,3-Glc	-	4AF9		[44]
		Galβ-1,3-Glc	E227D, Y228N	4AFC		[44]
		Galβ-1,3-GalNAc (T antigen)	-	4ASL		[44]
				4D3W		[52]
		Galβ-1,4-Glc (lactose)		4COU		[52]
		Glycerol	E227D, Y228N, D229N	4AFA		[44]
		Glycerol	R226I, E227G, Y228K	4AFB		[44]
	N-Epa6p	Galβ-1,4-Glc (lactose)		4COU		[52]
		Galβ-1,3-GalNAc (T-antigen)		4COW		[52]
		N-acetyl-D-lactosamine		4COY		[52]
		Lacto-N-biose		4COZ		[52]
		α1-3-galactobiose		4COV		[52]
	N-Epa9p	Galβ-1,4-Glc	-	4CP0		*
		Galβ-1,3-GlcNAc	-	4CP1		*
		Galβ-1,4-GlcNAc	-	4CP2		*
*S. cerevisiae*	N-Flo1p	Apo	-	4LHL	Cell-cell interaction via cell surface mannans	[39]
		Man	-	4LHK		[39]
	N-Lg-Flo1p	Apo	-	4GQ7	Cell-cell interaction via cell surface mannans and phosphomannans	[5]
		Manα-1,2-Man	-	4LHK		[39]
	N-Lg-Flo5p	Apo	-	2XJQ	Cell-cell interaction via cell surface mannans	[6]
		Man	-	2XJP		[6]
		Man_3_(D1)	-	2XJT		[6]
		Man_5_(D2-3)	-	2XJR		[6]
		Manα-1,2-Man	-	2XJS		[6]
		Manα-1,2-Man	S277A	2XJU		[6]
		Glc	-	2XJV		[6]
	N-Flo11p	Apo	-	4UYR	Cell-cell and cell-hydrophobic plastic adhesion via hydrophobic interactions	[64]
			-	4UYS		[64]
			-	4UYT		[64]

^1^ C-terminal end of human fibrinogen γ (Fg-γ): NH_3_-GEGQQHHLGGAKQAGDV-CO_2_. * Deposited in PDB but not yet published.

**Table 2:** Affinities (equilibrium dissociation constants K_D_) for the interaction between the yeast adhesins and their ligands.

Microorganism	Adhesin	Ligand	Dissociation Constant K_D_ (mM)	Refs
*C. albicans*	N-Als1p	Fibronectin	0.0016 ± 0.0006	[111]
		Laminin	0.013 ± 0.002	[111]
		Fucose	0.21 ± 0.03	[111]
		N-Als1p	0.020 ± 0.001	[111]
	N-Als3p	Fibronectin	0.41 ± 0.04	[112]
		Laminin	0.01 ± 0.001	[112]
		GlcNAc	0.034 ± 0.004	[112]
		N-Als3p	0.014 ± 0.002	[112]
*C. glabrata*	N-Epa1p	Galβ	0.115 ± 0.011	[43]
		Galβ-1,4-Glc ^1^	0.031 ± 0.004	[43]
			0.035 ± 0.006	[3]
		Galα-1,3-Gal ^2^	0.0050 ± 0.0009	[7]
		Galβ-1,3-Gal ^3^	0.0009 ± 0.00005	[7]
		Galβ-1,3-GalNAc ^4^	0.0035 ± 0.0004	[43]
			0.0021 ± 0.0003	[3]
			0.0009 ± 0.0001	[7]
		Galβ-1,3-GlcNAc ^5^	0.0049 ± 0.003	[7]
		Galβ-1,4-GlcNAc ^6^	0.0020 ± 0.0002	[7]
		Mucin	0.0047 ± 0.0009	[112]
		Fibronectin	0.911 10^−3^ ± 0.122 10^−3^	[8]
	N-Epa1p E227A	Fibronectin	0.317 10^−3^ ± 0.026 10^−3^	[8]
	N-Epa1p Y228W	Fibronectin	0.545 10^−3^ ± 0.058 10^−3^	[8]
	N-Epa6p	Galα-1,3-Gal ^2^	0.0004 ± 0.0007	[7]
		Galβ-1,3-Gal ^3^	0.0003 ± 0.0002	[7]
		Galβ-1,3-GalNAc ^4^	0.0005 ± 0.0011	[7]
		Galβ-1,3-GlcNAc ^5^	0.0148 ± 0.0001	[7]
		Galβ-1,4-GlcNAc ^6^	0.0094 ± 0.0013	[7]
	N-Epa7p	Galα-1,3-Gal ^2^	0.0124 ± 0.0009	[7]
		Galβ-1,3-Gal ^3^	0.0013 ± 0.0001	[7]
		Galβ-1,3-GalNAc ^4^	0.0015 ± 0.00001	[7]
		Galβ-1,3-GlcNAc ^5^	0.0147 ± 0.0004	[7]
		Galβ-1,4-GlcNAc ^6^	0.0048 ± 0.0004	[7]
*S. cerevisiae*	N-Flo1p	Man	8.7 ± 0.4	[39]
		Manα-1,2-Man	0.6 ± 0.1	[39]
		Manα-1,3-Man	3.3 ± 0.3	[39]
		Manα-1,6-Man	6.9 ± 0.6	[39]
		Glc	>100	[39]
	N-Lg-Flo1p	Man	0.8 ± 0.03	[54]
		Man	0.5 ± 0.05	[39]
		Man1P ^7^	0.06 ± 0.002	[54]
		Manα-1,2-Man	4.5 ± 0.38	[54]
		Manα-1,3-Man	3.9 ± 0.98	[54]
		Manα-1,6-Man	3.0 ± 0.33	[54]
		Glc	5.8 ± 0.80	[54]
		Glc1P ^8^	0.41 ± 0.03	[54]
	N-Flo5p (S227A)	Man	29.3 ± 3.6 (9.7 ± 0.6)	[6]
		Manα-1,2-Man	3.5 ± 0.3 (1.6 ± 0.1)	[6]
		Manα-1,3-Man	No binding	[6]
		Manα-1,6-Man	No binding	[6]
		Man_3_(D1)	2.8 ± 0.2	[6]
		Man_5_(D2-3)	2.2 ± 0.5	[6]
		Glc	>1000	[6]
	N-Flo11p	N-Flo11	0.0195 ± 0.0023	[64]

^1^ Lactose; ^2^ α1-3-galactobiose; ^3^ β1-3-galactobiose; ^4^ T-antigen; ^5^ lacto-N-biose; ^6^ N-acetyl-D-lactosamine; ^7^ Man1P: mannose 1-phosphate; ^8^ Glc1P: glucose 1-phosphate.

**Table 3:** Yeast adhesin–ligand interaction forces. Adapted from [121].

Cell type	Adhesin	Ligand	Rupture force	Comments	Refs
*C. albicans*	Als5p	Fibronectin	2800 ± 600 pN	SMFS ^1^ in vitro	[122]
*C. glabrata*	Epa6p	Hydrophobic surface (dodecane-thiol)	31–52 nN	SCFC ^2^: cell immobilization on the AFM probe using dopamine	[123]
*S. cerevisiae*	Flo1p	Flo1p	300 (100–600)	SMFS	[39]
*S. pastorianus*	Lg-Flo1p	Glucose	121 ± 53 pN	Amylose-coated AFM probe interact with cell surface	[124]

^1^ SMFS: single-molecule force spectroscopy; ^2^ SCFS: single-cell force spectroscopy.
